# GPI-anchored proteins are confined in subdiffraction clusters at the apical surface of polarized epithelial cells

**DOI:** 10.1042/BCJ20170582

**Published:** 2017-12-01

**Authors:** Simona Paladino, Stéphanie Lebreton, Mickaël Lelek, Patrizia Riccio, Sergio De Nicola, Christophe Zimmer, Chiara Zurzolo

**Affiliations:** 1 Department of Molecular Medicine and Medical Biotechnologies, University of Naples Federico II, Naples, Italy; 2 CEINGE Biotecnologie Avanzate, Naples, Italy; 3 Unité de Trafic Membranaire et Pathogénèse, Institut Pasteur, Paris, France; 4 Unité Imagerie et Modélisation, Institut Pasteur, UMR 3691 CNRS, C3BI USR 3756 IP CNRS, Paris, France; 5 CNR-SPIN Institute, National Research Council, Naples, Italy

**Keywords:** dSTORM, FLIM/FRET, GPI-anchored proteins, GPI-AP cluster modelling, plasma membrane organization, polarized epithelial cells, super-resolution microscopy

## Abstract

Spatio-temporal compartmentalization of membrane proteins is critical for the regulation of diverse vital functions in eukaryotic cells. It was previously shown that, at the apical surface of polarized MDCK cells, glycosylphosphatidylinositol (GPI)-anchored proteins (GPI-APs) are organized in small cholesterol-independent clusters of single GPI-AP species (homoclusters), which are required for the formation of larger cholesterol-dependent clusters formed by multiple GPI-AP species (heteroclusters). This clustered organization is crucial for the biological activities of GPI-APs; hence, understanding the spatio-temporal properties of their membrane organization is of fundamental importance. Here, by using direct stochastic optical reconstruction microscopy coupled to pair correlation analysis (pc-STORM), we were able to visualize and measure the size of these clusters. Specifically, we show that they are non-randomly distributed and have an average size of 67 nm. We also demonstrated that polarized MDCK and non-polarized CHO cells have similar cluster distribution and size, but different sensitivity to cholesterol depletion. Finally, we derived a model that allowed a quantitative characterization of the cluster organization of GPI-APs at the apical surface of polarized MDCK cells for the first time. Experimental FRET (fluorescence resonance energy transfer)/FLIM (fluorescence-lifetime imaging microscopy) data were correlated to the theoretical predictions of the model.

## Introduction

Glycosylphosphatidylinositol (GPI)-anchored proteins (GPI-APs) are abundant constituents of the plasma membrane of eukaryotic cells, where they play diverse vital functions, and display unique features being attached to the membrane external leaflet through a glycolipid anchor. Like for other membrane proteins, the spatio-temporal compartmentalization of GPI-APs proteins in the plasma membrane is critical for their proper function.

Several studies showed that in fibroblasts and in immune T cells, 20–40% of GPI-APs are organized in cholesterol-dependent nanoclusters containing 3–4 proteins of different species [1–4]. Recently, it has been shown that the organization of GPI-APs is very different in polarized epithelial cells where homoclusters form in the Golgi complex and appear to drive the formation of heteroclusters at the plasma membrane following polarized sorting to the apical domain [5]. Thus, unlike in fibroblasts, at the apical surface of polarized epithelial cells, GPI-APs are organized in small clusters of single GPI-AP species (homoclusters), which are independent of cholesterol [5]. These GPI-AP complexes, but not monomeric proteins, coalesce into larger cholesterol-dependent clusters formed by multiple GPI-APs species (heteroclusters) [5]. These data suggested that protein–protein and lipid–protein interactions might concur to the surface organization of GPI-APs. Furthermore, this clustered organization appears to be required to maintain the functional state of the protein at the apical membrane [5].

The molecular and spatio-temporal characteristics of GPI-AP clusters have been analyzed in fibroblastic cells using different single fluorescent molecule and super-resolution microscopy, and different models of the organization of GPI-APs have been proposed [6–9]. This analysis has proved to be much more demanding in polarized epithelial cells due to the complex organization of these cells in tall monolayers, which makes single-molecule imaging and super-resolution microscopy quite challenging. Here, for the first time to the best of our knowledge, we used super-resolution microscopy (direct stochastic optical reconstruction microscopy, dSTORM) coupled to pair correlation (PC) analysis [6,10] in polarized epithelial cells in order to investigate the spatial organization of GPI-APs at the apical surface in comparison with fibroblasts. Our data indicate that although GPI-AP clusters have similar size in both cell types, their requirements are different. Moreover, based on the current super-resolution analysis and using our previous FLIM (fluorescence-lifetime imaging microscopy) data [5], we built a theoretical model to explicate, in mathematical terms, the peculiar GPI-AP organization in polarized cells and to predict the energy transfer efficiency for heterocluster complexes in polarized cells.

## Experimental procedures

### Cell cultures, transfections and antibodies

MDCK cells were grown in DMEM (Sigma–Aldrich) containing 5% FBS. MDCK cells were co-transfected with sequences encoding for GFP-FR and mCherry-PLAP (placental alkaline phosphatase) or mCherry-p75 (refer to ref. [5]). MDCK cells transfected stably with mGFP-FR or transiently with mGFP-uPAR were previously described [5]. CHO cells were grown in HAM's F12 medium containing 10% FBS and were transfected with different cDNAs: cells stably expressing GFP-FR (kind gift of Dr S. Mayor, NCBS, Bangalore, India) were transiently co-transfected with mCherry-PLAP; CHO cells were transiently transfected with c-DNA encoding for PLAP.

We used the following antibodies: polyclonal anti-GFP (Clontech) and polyclonal anti-PLAP (from Rockland). We generated Fab fragments (using the protocol provided by Pierce) using either GFP or PLAP antibody and then they were coupled to CY5 dye (GE Healthcare Life Science).

### Modification of cholesterol content

To deplete cellular cholesterol content, cells were incubated with mevinolin (5 µM; Sigma–Aldrich) in DMEM supplemented with delipidated serum as previously described [5,11]. The low rate of cholesterol synthesis was allowed by supplementing cells with mevalonate in the culture medium (250 µM; Sigma–Aldrich).

The cells were loaded with cholesterol (10 mM) using water-soluble cholesterol-saturated methyl-β-cyclodextrin (Sigma–Aldrich), which was added to CO_2_-independent medium at 37°C for 40 min. To determine the rate of cholesterol depletion or addition, we measured cholesterol cellular levels by a colorimetric assay (Calbiochem) [5,11].

### STORM experiments

#### Microscopy system

STORM imaging was performed on a custom-built microscopy system featuring a Nikon Ti-E eclipse microscope body, a 647 nm laser (MPB Communications) and a 405 nm laser (Oxxius) for wide-field illumination and an EMCCD camera (Andor IXON 897 ultra). The microscope is equipped with a Perfect Focus System (PFS; Nikon) to prevent axial drift of the sample. Micromanager is used to control the microscope and the camera during image acquisition. The laser power is adjusted during image acquisition by controlling the AOTF (AA Optoelectronic) using a Python program [12].

#### Sample preparation

Polarized MDCK or CHO cells were fixed with 4% paraformaldehyde and 0.02% glutaraldehyde for 20 min to prevent protein diffusion. After quenching with NH_4_Cl for 10 min and saturation with gelatin at 0.2% for reducing non-specific antibody bindings, cells were stained with the Fab-GFP or Fab-PLAP coupled to the dye Cy5 for 45 min. In the sets of experiments, to calibrate our experimental procedure, Fab-GFP was diluted one-tenth, while it was undiluted (2 μg/ml) for the rest of experiments.

Because the polarized cells are growing on filter, a specific mounting was needed for high-resolution microscopy. Multi-well slides with eight wells (MP Biomedicals, LLC) were used to keep the cells sufficiently close to the coverslip, considering the working distance of the objective lens. The filters were cut and each piece matched the size of the hole engraved in the cover glass. These holes were filled with an oxygen scavenger buffer, as previously described [13], which promotes fluorophore blinking when in contact with the filter and the cells. Fluorescent beads were added to the sample before mounting in order to allow estimation and computational correction of spatial drifts.

#### Imaging

Because the polarized cells were grown on the filter placed between coverslips, the apical surface of cells is not always close to the coverslip, making STORM imaging more difficult. To find polarized cells and avoid the axial drift during STORM acquisition, the coverslip is scanned along the *x*- and *y*-axes with the PFS turned on until cells are found sufficiently close to the coverslip and in the offset range of the PFS. PALM/STORM imaging is performed as previously described [12]. Image acquisition parameters are as follows: EM camera gain 300; exposure time 100 ms; binning 1 : 1; in each experiment, a sequence of 30 000–50 000 full-sized raw images (512 × 512 pixels) was acquired. Raw images were processed to compute molecular localizations using PALMTT, a modified version of the Matlab-based single particle tracking software MTT [14]. Another in-house Matlab program, PALM vis, was used to correct for sample drift and generate super-resolution visualizations.

### Pair correlation-photo-activated localization microscopy analysis

The computed localization data were further subjected to a PC analysis by adapting the previously described procedure [15]. Normalized pair-wise correlation functions *g*(*r*) were computed from the localization data and then fitted to each of the following two equations:

1 $$g^{\mathrm{random}}(r)=\frac{1}{4\pi \sigma_{s}^{2}\rho }\exp\left(-\frac{r^{2}}{4\sigma_{s}^{2}}\right)+1$$

and

2 $$g^{\mathrm{cluster}}(r)=\frac{1}{4\pi \sigma_{s}^{2}\rho }\exp\left(-\frac{r^{2}}{4\sigma_{s}^{2}}\right)+\left(1+A\;\exp\left(-\frac{r}{\xi }\right)\right)*\frac{1}{4\pi \sigma_{s}^{2}}\exp\left(-\frac{r^{2}}{4\sigma_{s}^{2}}\right)$$

Eqn (1) is the correlation function expected for a random distribution of isolated (non-clustered) molecules, where *σ*_s_ is the standard deviation of random localization errors and *ρ* is the density (number of localizations per unit surface). Eqn (2) is the correlation function expected for clustered molecules, assuming that the pair correlation of protein localizations within a cluster obeys $g^{\mathrm{protein}}(r)=1+A\;\exp(-(r/\xi ))$, where *ξ* measures the cluster size and *A* is the amplitude of the protein correlation extrapolated to distance *r* = 0. Fitting eqn (1) to pair correlation functions obtained for isolated molecules yields an estimate of *σ*_s_ (along with *ρ*), which is then held constant when fitting eqn (2). The latter fitting thus yields three parameters: *A*, *ρ* and *ξ*.

**Figure 1.**
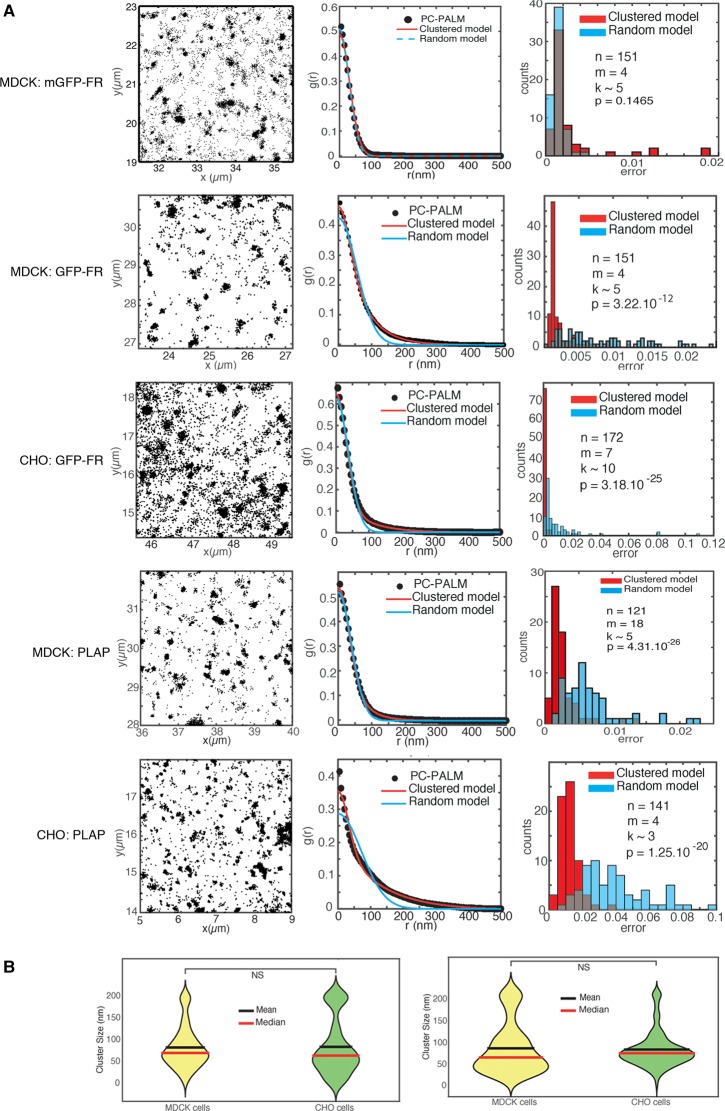
GPI-APs are clustered at the plasma membrane (apical surface) of MDCK and CHO cells. (**A**) Leftmost column: panels show representative images (4 × 4 µm area) of STORM localizations of mGFP-FR, GFP-FR and PLAP at the plasma membrane of MDCK (apical surface) and CHO cells. The mean and standard deviation of single-molecule localization numbers *N* for the *n* regions of interest analyzed is as follows: *N* = 23 921 ± 27 624 for mGFP-FR, *N* = 17 508 ± 15 487 for GFP-FR, *N* = 52 680 ± 51 642 for PLAP in MDCK cells and *N* = 33 355 ± 22 938 for GFP-FR, *N* = 54 397 ± 22 938 for PLAP in CHO cells. Middle column: the pair correlation (pc-PALM) function computed from the areas shown in the left panels. Black dots are experimental data, blue and red curves are, respectively, the random model and clustered model fitted to the data (see Experimental procedures). Rightmost column: histograms show the distribution of mean squared difference between the data and the two fitted models for *n* distinct regions of 4 × 4 µm obtained from *m* experiments with an average of *k* cells per experiments. The blue histogram shows the difference between the data and the random model, and the red histogram shows the difference between the data and the clustered model. Overlaps between the two histograms appear in grey. A Kolmogorov–Smirnov test is used to compare the two error distributions and to assess if the clustered model provides a significantly better fit than the random model, with the *P*-value as indicated. If the difference is not significant (*P* > 0.05), we categorized the molecular distribution as random. While for mGFP-FR, the random and clustered models provide an equally good fit (*P* = 0.15), for GFP-FR the clustered model fits the data significantly better than the random model (*P* < 10^−14^) both in MDCK and CHO cells. Note that these *P*-values were computed from the residuals of the fits *n* > 100 regions of interest in each case. (**B**) Violin plots show the distribution of GFP-FR and PLAP cluster sizes as obtained from the clustered model fits (mean and median are indicated).

Squared regions of 4 × 4 μm on the cell membrane were manually selected on the basis of STORM localization images (e.g. Figure 1A). For each area, the pair-wise correlation function of these localizations was computed and then fitted to the random and clustered models (e.g. Figure 1B). For each PC curve, we computed the mean squared error of the residual, i.e. the difference between the data and each of the two fitted models. The distribution of errors for the random and the clustered model obtained from many 4 × 4 μm regions were each represented by a histogram (e.g. Figure 1C). The two distributions were then compared using a Kolmogorov–Smirnov test in order to determine if the clustered model fits the data significantly better than the random model. If this difference was not significant, we categorized the molecular distributions as random and further analyzed the distribution of fitted *σ*_s_ (see Supplementary Figure S1). Otherwise, we categorized it as clustered and used the fitted parameters *ξ* to determine the cluster sizes. Simulation of random and clustered molecular distribution is shown in Supplementary Figure S2.

For each experiment, the *P*-value was computed on the residuals of the fits of more than 100 4 × 4 μm regions. Moreover, the analysis was performed at the same labelling densities and the comparable number of localizations was considered for each STORM dataset.

### GPI-AP cluster modelling

#### Comparison of the model of randomly distributed donors and acceptors with the experimental FLIM data

Our experimental data indicate that GPI-APs are organized in clusters in epithelial polarized cells [5], thus first we compared our experimental FLIM data with a model of random distribution of donors and acceptors.

In a random planar array of donors and acceptors in which (1) there is no transfer energy between donors, (2) the number of acceptors in the excited state is smaller than that in the ground state, (3) the distance between donors and acceptors does not change during the excited state lifetime of the donor and (4) the Forster radius is the same for all donor–acceptor pairs, and the rate of transfer is the sum of the rate of energy transfer for all donor–acceptor pairs.

In the model of randomly distributed donors and acceptors, the efficiency of energy transfer is governed by three parameters: the Forster radius, the surface density of acceptors, and the distance of closest approach of donor and acceptor.

The basic prediction of this model is that as the surface density of acceptor increases, the average lifetime of the donors decreases and the fluorescence intensity decay deviates from a simple single exponential because of the increasing energy transfer. Furthermore, the energy transfer efficiency goes to zero at low acceptor surface densities and it tends to zero more slowly with the increase of the distance of closest approach of donor and acceptor (the slope of the curve of energy transfer efficiency vs. density of acceptors decreases). Our FLIM data showing that energy transfer is independent of donor and acceptor densities (Figure 4A and [5]) do not fit with the random model. Indeed, we found energy transfer also at low concentrations of donors or acceptors and it never goes to zero as in the case of a model of random distribution of donors and acceptors.

#### Model of spatial organization of heteroclusters

On the basis of the experimental data provided by N&B (Number and Brightness) and FLIM analysis [5], we derived a simple model, which would predict/describe the spatial organization of heteroclusters based on the estimate of the energy transfer efficiency of heterocluster structures.

Because the energy transfer between GPI-APs occurs (i) on whole apical surface of MDCK cells and (ii) only when homoclusters are present [5], we consider a random distribution of heteroclusters in which a single heterocluster is a dimer formed by two homoclusters at distance *R*, one labelled by a donor (hD) and the other by an acceptor (hA) (Figure 4B). In this model, donors and acceptors are localized in the volumes of the two homoclusters.

From the FLIM data, we know that only donor–acceptor-labelled heteroclusters are in FRET (fluorescence resonance energy transfer) and the percentage of interacting donor is independent of both donor and acceptor fluorescence intensities and of the ratio D : A of their fluorescence intensities [5]. Following the approach developed by Padilla-Parra et al. [16,17], we have quantified the minimal percentage of donor $m_{\mathrm{fD}}$ engaged in FRET from the recovered lifetime ⟨τ⟩ of GFP-FR in the presence of mCherry-PLAP. Specifically, in a two-component system in which a fraction of donor undergoes FRET and a fraction does not, $m_{\mathrm{fD}}$ is determined by the minimization of the fraction of donors involved in FRET: $f_{\mathrm{DA}}=N_{\mathrm{DA}}/\left(\;N_{D}+N_{\mathrm{DA}}\right)$ where $N_{\mathrm{DA}}$ is the number of donor–acceptor complexes and $N_{D}$ is the number of free donors. In this case, it can be shown that the minimal percentage of donor can be written in terms of the measured mean lifetime ⟨τ⟩ in the following form:

3 $$m_{\mathrm{fD}}=\frac{\left(1-\langle \tau \rangle /\tau_{D}\right)}{{\left(1-\langle \tau \rangle /2\tau_{D}\right)}^{2}}$$

where $\tau_{D}=2.47\;\mathrm{ns}$ is the fixed lifetime donor value extracted from cells expressing GFP-FR alone (Supplementary Figure S8 and [5]). According to the Forster theory, the FRET efficiency $E_{\mathrm{DA}}$ varies inversely as the sixth power of the distance between the donor and acceptor

4 $$E_{\mathrm{DA}}=\frac{R_{0}^{6}}{R^{6}+R_{0}^{6}}$$

where *R* is the distance of separation between the donor and the acceptor and the Forster distance *R*_0_ is defined as that separation for which the energy transfer efficiency is 50% and is calculated by the following expression:

5 $$R_{0}(Å)=(9.79\times 10^{3})[Q_{0}Jk^{2}n(c{)}^{-4}{]}^{1/6}$$

In eqn (5), $Q_{0}$ is the quantum yield (0.64 for EGFP) [18] of the donor without the acceptor, *J* is the spectral overlap integral, *n*(*c*) is the concentration-dependent refractive index of the interposed medium and $k^{2}$ is the orientation factor and *J* is the spectral overlap integral which depends on the relative orientation of the two dipole moment vectors.

In general, this orientation factor can vary from 0 to 4 owing to different donor/acceptor orientation distributions. Uncertainties in the value of $k^{2}$ become critical in membrane systems because their intrinsic anisotropy can lead to a significant deviation from the average value $\langle k^{2}\rangle =2/3$ corresponding to a uniformly random orientation of the donors and acceptors [19,20]. A change in $k^{2}$ results in a change of the Forster radius *R*_0_ that in turn affects the transfer efficiency as predicted by eqn (2). The Foster theory allows making a direct connection of the transfer efficiency rate *E* to the experimentally measurable lifetime donor ⟨τ⟩ via the simple expression

6 $$E=1-\frac{\left\langle \tau \right\rangle }{\tau_{D}}=\langle E_{\mathrm{DA}}(R,R_{0})\rangle$$

$\left\langle E_{\mathrm{DA}}(R,R_{0})\right\rangle$ is the average value of the energy transfer efficiency of a single donor–acceptor-labelled heterocluster that it could be written in this form to highlight its dependence on the Forster distance *R*_0_ and the distance *R* between donor–acceptor pairs.

In eqn (6), the average value of the energy transfer efficiency is taken over the spatial distribution of the positions of the pair in FRET and depends on the spectral properties of the pair in FRET and, ultimately, on the spatial structure of the two homoclusters.

In our model, the relative orientation of the donor and acceptor dipoles is accounted for implicitly through the dependence of the Foster distance $R_{0}$ on the orientation factor. The model was fitted to our FRET data taking the value of $R_{0}$ obtained by the spectral measurement for EGFP donor and mCherry acceptor [21].

From eqn (5), it can be seen that the Foster distance decreases with the increase of the refractive index of the surrounding medium as $n(c{)}^{-2/3}$, which means that the energy transfer efficiency depends also on the properties of the medium interposed between the donor and the acceptor. This is of fundamental importance because we have demonstrated that heteroclusters are cholesterol-dependent and lost upon cholesterol depletion [5].

We consider a simple model in which the homoclusters hD and hA are taken as planar thin cylindrical structures separated by a distance *d* (Figure 4B). These structures represent the protein portion in FRET.

On the basis of N&B data [5], we know that each homocluster is mainly composed of 3–4 monomers. Since both GFP and mCherry have similar diameter [22–24] (2.5–3.0 nm) and it is unlikely that GPI-APs form linear clusters because protein–protein and protein–lipid interactions regulate GPI-AP oligomerization [5,11,25] and taking into account that phospholipid and/or cholesterol molecules could be present between GPI-APs, we assume that the cylindrical shape of each homocluster has a base circle whose diameter *l* is of the order of 6–10 nm. Moreover, on the basis of crystallographic data showing that the GFP/mCherry β-barrel is 4 nm and chromophore is located roughly in the centre and considering that protein can undergo small oscillations along the *Z*-axis (e.g. because of interactions with lipid environment, with other proteins), we can assume that the minimal thickness of the cylinder structure is ∼1–1.5 nm.

According to the geometry schematically depicted in Figure 4B, the distance *R* between donor–acceptor-labelled homoclusters can be written in terms of the Cartesian co-ordinates (*x*_D_, *y*_D_, *z*_D_) of the position of the donor inside the volume $V_{\mathrm{hD}}$ of the homocluster hD and the co-ordinates (*x*_A_, *y*_A_, *z*_A_) of the position of the acceptor inside the corresponding volume $V_{\mathrm{hA}}$

7 $$R={\left|{\left(x_{A}+l+d-x_{D}\right)}^{2}+{\left(y_{A}-y_{D}\right)}^{2}+{\left(z_{A}-z_{D}\right)}^{2}\right|}^{1/2}$$

The expression for the efficiency of energy transfer $\left\langle E_{\mathrm{DA}}(R,R_{0})\right\rangle$ of the heterocluster can be written in terms of the weighted probability of Forster law as an integral over the volumes of the two homoclusters. If we assume that the positions of the donors and acceptors are distributed uniformly and independently inside the respective volumes, we can write the average value of the transfer efficiency

8 $$\left\langle E_{\mathrm{DA}}(R,R_{0})\right\rangle =\frac{1}{V_{\mathrm{hA}}V_{\mathrm{hD}}}\int_{V_{\mathrm{hA}}}\int_{V_{\mathrm{hD}}}\frac{R_{0}^{6}}{R_{0}^{6}+R^{6}}d^{3}r_{A}d^{3}r_{D}$$

where the distance *R* between donor and acceptor is given by eqn (7). Eqn (8) expresses the spatial averaging of the transfer efficiency as a six-dimensional integration over the positions of the pair in FRET in the respective homoclusters. It can be related by eqn (6) to the experimentally measured energy transfer efficiencies.

In Figure 4A, FRET efficiency derived from experimental data (Supplementary Figure S8 and ref. [5]) is plotted as a function of the donor-to-acceptor ratio D : A. The solid line is a nonlinear least square fit of the experimental measurements of transfer efficiency using eqns (6–8). The fit was performed with the Levenberg–Marquardt algorithm assuming Foster radius *R*_0_ = 5.24 nm and equal volume homoclusters hA and hD $V_{\mathrm{hA}}=V_{\mathrm{hD}}=\pi l^{2}h/4$ with equal size *l* *=* 8 nm and thickness *h* *=* 1 nm.

The fit analysis allows us (i) to recover the distance (*d*) between the two homoclusters and (ii) estimate the overall size (*D*) of the heterocluster as *D* = 2*l* + *d*, i.e. the length of the side of the two homoclusters plus the distance *d* between them. From the fit, we determined the distance between the two homoclusters *d* *=* 1.4 ± 0.1 nm and the size of the heterocluster *D* = 17.4 ± 0.1 nm. Moreover, the model allows exploring the influence of the geometry on the overall transfer efficiency *E* (eqn 6) and calculating the total length of heterocluster dependence on the size of the composing objects.

### Statistical analysis

Kolmogorov–Smirnov tests are used to determine if the data are better fit by a clustered than a random model (see above), and Wilcoxon tests are computed to compare distributions of cluster sizes between different conditions.

## Results

### Super-resolution analysis of GPI-APs clusters at the apical surface of MDCK cells

Characterizing the homocluster organization of GPI-APs by imaging is challenging because current data indicate that the size of membrane protein clusters is typically <100 nm, well below the resolution of conventional microscopy. Here, we used super-resolution microscopy by single-molecule localization (dSTORM) [6,15,26–28], which achieves resolutions of ∼20–50 nm. The molecular distribution of the proteins obtained by dSTORM was further subjected to PC analysis (pc-STORM) [15], which can reveal whether the protein is randomly distributed or clustered and, in the latter case, estimate the typical cluster size (see Experimental procedures and Supplementary Figures S1 and S2).

Using this approach, we analyzed the characteristics of clusters formed by various apical GPI-proteins stably transfected in MDCK cells. Specifically, for GPI-AP cluster model proteins, we chose GFP-FR, a chimeric protein used before by us and others [1,4–6,25], in which the GFP moiety is fused to the GPI anchor attachment signal of the Folate Receptor. We used two chimeric forms of GFP (mGFP-FR and mGFP-uPAR) as control monomeric proteins, in which GFP is fused to urokinase-type plasminogen-activated receptor, that were previously shown to exist exclusively in the monomeric state [5]. We analyzed the behaviour of p75-GFP as model transmembrane protein (where the p75-NTR receptor transmembrane domain and cytosolic tail are fused to GFP) [11].

To estimate the resolution of our STORM images, and as control of our experimental procedure, we used diluted anti-GFP antibody fragments (Fab) to target our four model proteins. In these conditions, because of the dilution, we expect to see single isolated fluorescent molecules even if the proteins are clustered. Indeed, the pair correlation functions were well fitted by a model assuming randomly distributed single molecules with a localization precision of *σ*_s_ = 20–30 nm (corresponding to an estimated STORM resolution of ∼50–70 nm) for all our model proteins (Supplementary Figure S1). Consistently, a model assuming protein clustering did not significantly improve the quality of these fits (Supplementary Figure S1). In contrast, when using undiluted antibodies, the pair correlation for GFP-FR was no longer fitted by the random model (Figure 1A), whereas for the monomeric GPI-AP (mGFP-FR and mGFP-uPAR) the random model provided a good fit (Supplementary Figure S1 and Figure 1A). Instead, the pair correlation for GFP-FR was much better fitted by a model assuming clusters, in which the proteins are exponentially distributed (and assuming the above localization precision *σ*_s_) (Figure 1A). This clearly indicates that at the apical surface of polarized MDCK cells, GFP-FR proteins are clustered. Moreover, PC analysis showed that GFP-FR is clustered also at the plasma membrane of CHO fibroblasts (Figure 1A). This was consistent with earlier measurements for GPI-AP in non-polarized cells, further validating our approach [15]. Next, we measured the size of the clusters in the two different cell lines and found that their size was similar (72 and 66 nm in MDCK and CHO cells, respectively; Wilcoxon rank sum test *P* = 0.22) (Figure 1B). We also verified that the cluster sizes in the MDCK GFP-FR control experiment were similar when considering either the whole set of images (30 000 frames) or the 10 000 first or last frames (Supplementary Figure S3), thus ruling out the possibility that residual GPI-AP diffusion during STORM imaging significantly affected cluster size measurements.

Furthermore, these data were corroborated by the analysis of a native apical GPI-AP, PLAP. PC analysis indicated that these proteins are also clustered with a cluster size of 67 nm for MDCK cells and 75 nm for CHO cells (*P* = 0.1 from a Wilcoxon test), thus very similar to the sizes determined for GFP-FR (Figure 1A,B). Temporal colour coding of the localizations further indicated clustering of molecules in PLAP, as opposed to the occurrence of predominantly isolated proteins for p75-NTR (Supplementary Figure S4).

Overall, these data confirm the size values found by others for different GPI-AP clusters in fibroblastic cells [15] and reveal that GPI-APs have a similar cluster size in polarized MDCK cells. Because transmembrane or prenylated proteins have been found in larger clusters (up to 120 nm) [15], our observations also suggest that the size of these confinement areas might be an intrinsic characteristic of GPI-APs, possibly linked to the GPI anchor properties.

### Super-resolution analysis of GPI-AP clusters upon cholesterol perturbation in MDCK and CHO cells

**Figure 2.**
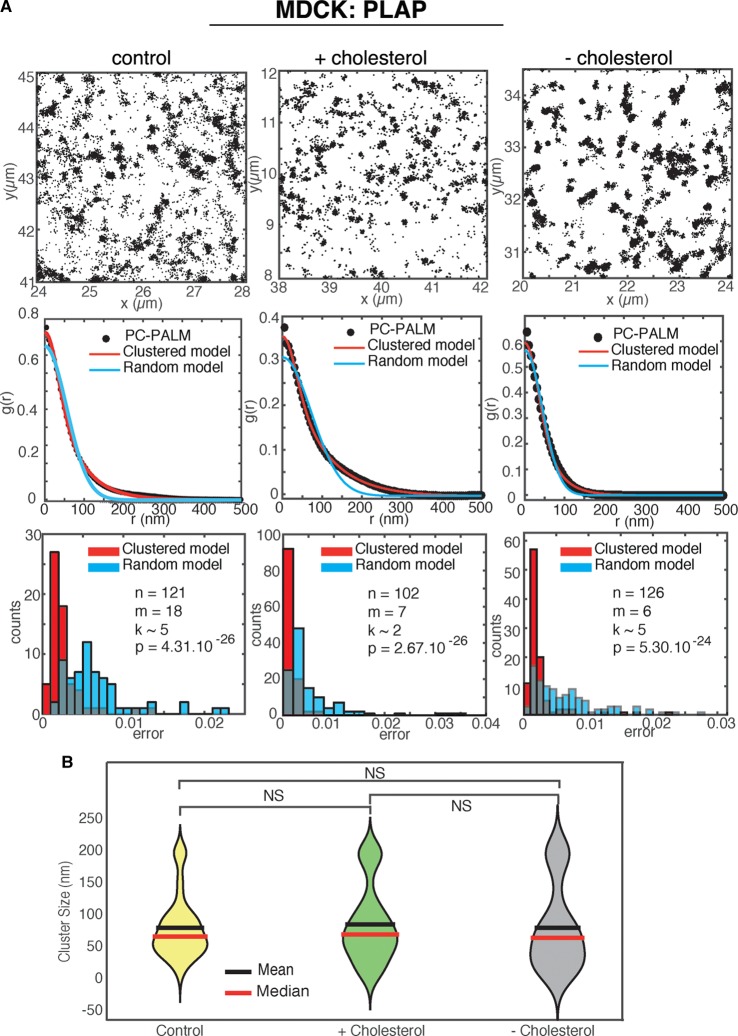
Cholesterol perturbation does not affect the spatial distribution of PLAP at the apical surface of MDCK cells. (**A**) Top panels: representative images (4 × 4 μm area) of STORM localizations of PLAP in control conditions and upon cholesterol modification (either addition or depletion) at the apical membrane of MDCK cells are shown. The mean and standard deviation of single-molecule localization numbers *N* for the *n* regions of interest analysed are as follows: *N* = 52 680 ± 51 642 for control condition, *N* = 75 183 ± 68 355 for cholesterol addition, and 59 321 ± 53 349 for cholesterol depletion. The pair correlation function (middle panels) and the distribution of mean squared errors between the data and the fitted model (lower panels) are shown as in Figure 1. Both in untreated and in treated cells, the pair correlation data are fitted significantly better by a clustered model compared with a random model (*P* < 10^−24^), revealing a clustered organization of PLAP in all conditions. (**B**) The distribution of calculated PLAP cluster sizes is shown as violin plot, with mean and median as indicated.

**Figure 3.**
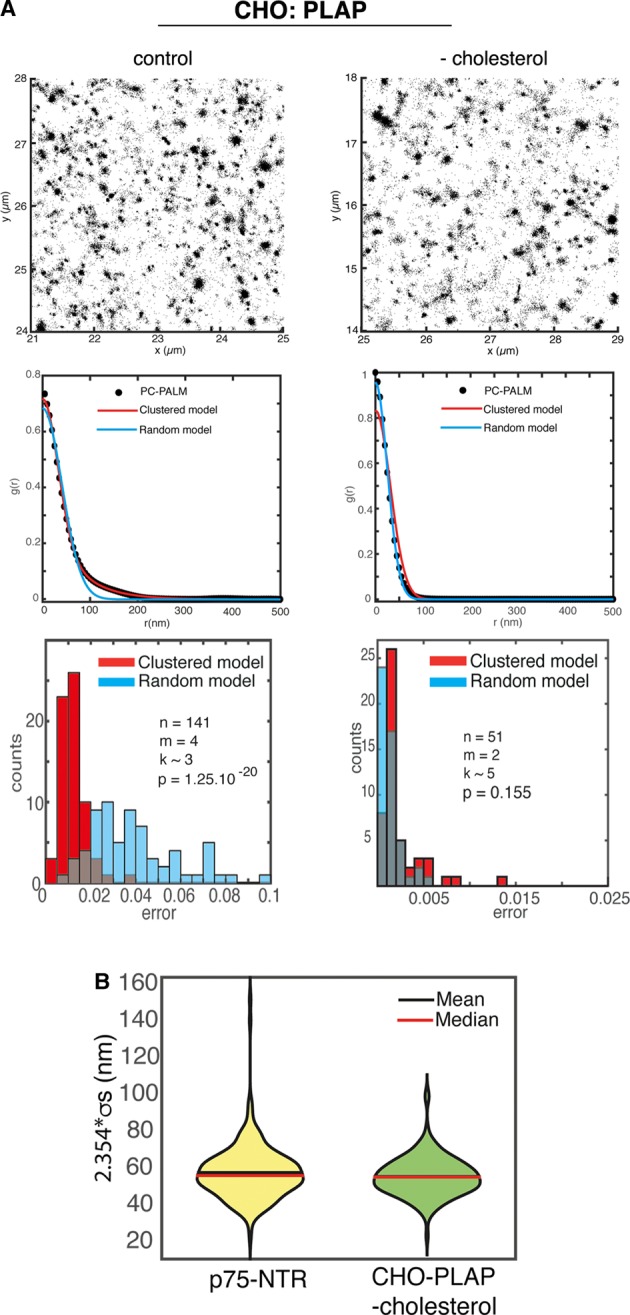
Cholesterol depletion affects the PLAP organization at the surface of CHO cells. (**A**) Top panels: representative images (4 × 4 μm area) of STORM localizations of PLAP in control conditions (left) and upon cholesterol depletion (right) at the surface of CHO cells. The mean and standard deviation of single-molecule localization numbers *N* for the *n* regions of interest analyzed are 54 397 ± 22 938 and 96 544 ± 56 281 in control conditions and upon cholesterol depletion, respectively. Middle and bottom panels: PC-PALM analysis results of PLAP are shown as in Figure 1. In the control cells, the pair correlation data are fitted significantly better by a clustered model compared with a random model (*P* < 10^−17^), revealing a clustered organization of PLAP. Upon depletion of cholesterol, both models fit the data equally well (*P* = 0.155), suggesting that PLAP becomes randomly organized. (**B**) Violin plots show the distribution of 2.354 * *σs* obtained by fitting the random model to all 4 × 4 μm regions in CHO cells expressing PLAP upon cholesterol depletion and in MDCK cells expressing p75-NTR using the diluted fab-GFP (see also Supplementary Figure S1).

Previous studies showed that cholesterol has a fundamental role in the membrane organization of GPI-APs both in fibroblasts and in epithelial cells, but at different spatial scales [3–5,11,25]. Therefore, we applied STORM and PC analysis to investigate the effects of cholesterol perturbation (addition or depletion) on the spatial distribution of PLAP in polarized MDCK cells at high resolution. We found that at the apical surface of polarized MDCK cells, PLAP remained clustered upon either cholesterol depletion (Figure 2A, right panels) or addition (Figure 2A, middle panels), indicating that in polarized cells PLAP clusters are cholesterol-independent. Consistently, the size of the clusters was not affected by these treatments (Figure 2B and Table 1). These data are in line with previous analyses showing the lack of sensitivity to cholesterol depletion of GPI-AP homoclusters in polarized epithelial cells [5,29]. As a positive control, we monitored the behaviour of PLAP in CHO cells upon cholesterol depletion. Although after mevinolin treatment we detected comparable reduction in total cholesterol in MDCK and CHO cells (40–45%, Supplementary Figure S5), unlike MDCK cells, PLAP no longer formed clusters and assumed a random organization in CHO cells upon this treatment (Figure 3A). When fitting the PC-PALM curves for PLAP in cholesterol-depleted CHO cell curves with a random model, we obtained a distribution of localization precision *σ*_s_ similar to that for p75-NTR in MDCK cells when using diluted fab-GFP (Figure 3B and Supplementary Figure S1), consistent with a random organization of these molecules on the membrane. Reanalysis of these data using a recently proposed temporal accumulation method led to similar conclusions (Supplementary Figure S6) [30,31].

**Table 1:** Role of cholesterol in PLAP organization at the apical surface of MDCK cells using pc-STORM

*P*-value	Control 18exp/121 PC-PALM curves	Cholesterol depletion 7exp/102 PC-PALM curves	Cholesterol addition 6exp/126 PC-PALM curves
Control	X	0.374	0.779
Cholesterol depletion	0.374	X	0.625
Cholesterol addition	0.779	0.625	X

Number of experiments and PC-PALM curves are shown together with the relative statistical analysis.

Moreover, the exogenous addition of cholesterol did not affect the spatial distribution of PLAP, which remained clustered in CHO cells (Supplementary Figure S7). Interestingly, PLAP cluster size was unaltered in these conditions (Supplementary Figure S7), indicating that increased levels of cholesterol do not affect the spatial distribution of GPI-AP clusters. Overall, these data indicate that in CHO cells, cholesterol is required to maintain a clustered organization of GPI-AP at the cell surface, but that increasing cholesterol beyond physiological levels did not promote the coalescence of GPI-AP cluster into larger clusters.

Finally, quantitative analysis of single-molecule localizations in polarized epithelial cells revealed that GPI-AP clusters have similar size as in fibroblasts, but different sensitivity to cholesterol depletion, supporting a different organization at the plasma membrane in the two cell types [29,32].

### GPI-AP cluster modelling in polarized epithelial cells

We have previously shown that at the apical surface of polarized MDCK cells, GPI-APs display two levels of organization: cholesterol-independent homoclusters of the same GPI-AP species and cholesterol-dependent heteroclusters derived by the coalescence of homoclusters [5].

To gain more insights into the molecular organization of GPI-APs in polarized epithelial cells, we developed a theoretical model to depict the spatial organization of GPI-AP clusters in polarized MDCK cells, based on estimate of the energy transfer efficiency of heteroclusters (see also Experimental procedures).

**Figure 4.**
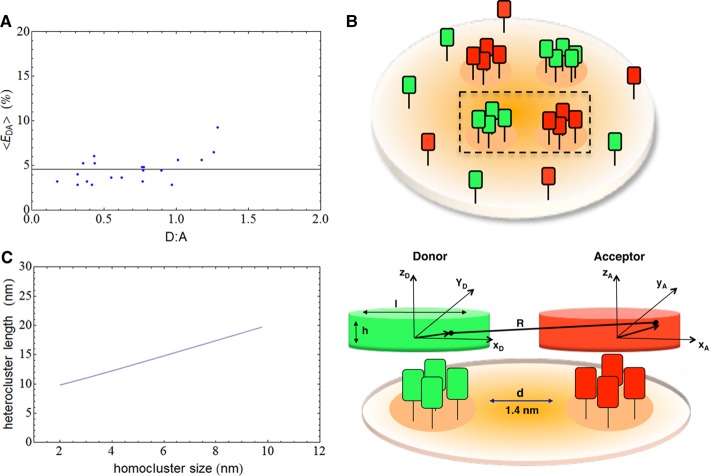
Spatial organization of GPI-AP heteroclusters at the apical surface of MDCK cells. (**A**) The energy transfer efficiency is plotted in the graph as a function of the ratio D : A of the donor-to-acceptor fluorescence intensities. The data of energy transfer efficiency (blue dots) are obtained from measured mean lifetime $\left\langle \tau \right\rangle$ of GFP-FR in the presence of mCherry-PLAP (Supplementary Figure S8) and are displayed for different values of the ratio D : A. The solid line is the fit of the experimental data calculated according to the heterocluster model assuming two homoclusters of size 8 nm and taking the Forster radius *R*_0_ = 5.24 nm. (**B**) The scheme depicts the geometry of heterocluster derived by a simple model of spatial organization of heteroclusters. Each heterocluster is a dimer formed by two homoclusters one labelled by a donor (hD) and the other by an acceptor (hA) at distance *R*. In this model, we assume cylindrical homoclusters hD and hA of diameter *l* separated by distance *d*. This distance is fitted to experimental FRET data to recover the overall size of heterocluster structure. *R*: donor–acceptor distance; *d*: distance between homoclusters; *l*: diameter of homocluster; *x*_D_, *y*_D_, *z*_d_ and *x*_A_, *y*_A_, *z*_A_: Cartesian co-ordinates of the position of donor and acceptor, respectively. (**C**) The graph shows that the length of the heterocluster is function of the size of the homocluster calculated for Forster radius *R*_0_ = 5.24 nm.

Using the FRET/FLIM approach, we determined that there is energy transfer between two GPI-APs, GFP-FR and mCherry-PLAP, at the apical surface of polarized epithelial MDCK cells (Supplementary Figure S8 and [5]). Moreover, our FRET–FLIM data showed that energy transfer is independent of donor and acceptor densities. Indeed, from our previous measurement of the mean lifetime $\left\langle \tau \right\rangle$ of GFP-FR in the presence of mCherry-PLAP (Supplementary Figure S8 and [5]) and using eqn (6), we calculated the energy transfer efficiency as a function of the ratio of the donor-to-acceptor fluorescence intensities (Figure 4A). The data show energy transfer efficiency also at low concentrations of donors and acceptors in disagreement with a random organization of donors and acceptors (see also Experimental procedures). Furthermore, it was found that energy transfer between two different GPI-APs occurs (i) on the whole apical surface of MDCK cells and (ii) only when homoclusters are present and (iii) between homoclusters, but not between monomers or between homoclusters and monomers [5]. In view of these findings, we developed a model that, although its simplifying assumptions, provides a convenient framework to analyze the molecular organization of GPI-APs on the apical surface of MDCK cells. The model assumes a random distribution of heteroclusters on the apical surface where a single heterocluster behaves as a dimer formed by two homoclusters located at FRET distance (*d*). Figure 4B depicts the considered geometry schematically. The heterocluster is formed by the homocluster (hD) labelled by a donor and the homocluster (hA) labelled by an acceptor located at distance (*R*). Donors and acceptors are localized inside the respective volumes of the two homoclusters, which are assumed to be planar structures in the shape of thin cylinders with equal size (*l*) and thickness (*h*) (Figure 4B and Experimental procedures). The cylindrical geometry has been considered based on the β-barrel structure of our model proteins (GFP-FR and mCherry-PLAP, for instance) [22–24] attached through the GPI anchor to the external leaflet of the plasma membrane, and having a limited volume of interaction.

We proceeded to estimate the size of the heteroclusters by determining the distance between the homoclusters that allows to best fit the experimental FRET–FLIM data (Figure 4B and Experimental procedures). It is noteworthy that this distance is crucially dependent on the size of the homoclusters as well as on the Forster radius governing molecular energy transfer (see also Experimental procedures). From our N&B data [5], we know that each homocluster is mainly composed of 3–4 monomers. Taking into account that (i) both GFP and mCherry have similar diameters of 2.5–3 nm [22–24] and (ii) that GPI-APs unlikely form linear clusters since protein–protein and protein–lipid interactions regulate GPI-AP oligomerization [5,11,25], we assume that the cylindrical shape of the homocluster has a base circle whose diameter l is of the order of 6–10 nm (considering only the space occupied by proteins or taking into account also the space occupied on average by phospholipids and/or cholesterol, respectively). Moreover, based on crystallographic data showing that the length of GFP/mCherry β-barrel is 4 nm and the chromophore is roughly located at the barrel centre [22–24] and taking into account that proteins can undergo slight excursions along the *Z*-axis (e.g. because of interactions with lipid environment, with other proteins), we assume that the minimal thickness (*h*) of the cylinder structure is 1–1.5 nm.

Under these assumptions, the model predicts non-zero energy transfer efficiency even at low donor/acceptor fluorescence intensities ratio (Figure 4A). This is in good agreement with the experimental data and in contrast with the predictions for randomly distributed donor and acceptor molecules (Experimental procedures). Thus, by fitting FRET–FLIM experimental data with this model, we have estimated the distance between homoclusters (*d*) and the overall size (*D*) of heterocluster as *D* = 2*l* + *d* (comprising the length of the diameter of the two homoclusters plus the distance *d* between them; Figure 4B and Experimental procedures). On the basis of these considerations, assuming that heterocluster is formed by two cylindrical homoclusters of equal size (*l* = 8 nm; *h* = 1 nm), we calculated that the distance between homoclusters is *d* = 1.4 ± 0.1 nm and the size of the heterocluster is *D* = 17.4 ± 0.1 nm. According to this model, the length of the heterocluster increases with the increase of the size of the homocluster (Figure 4C). In particular, the plot in Figure 4C shows the calculated lengths of the heterocluster as function of homocluster size in the range of 2–10 nm on the basis of the aforementioned protein size estimates.

## Discussion

Spatio-temporal organization of GPI-APs is crucial for regulating their biological activities at the plasma membrane. Several studies showed that GPI-APs are organized in small clusters at the surface of different cell types [1–5,7]. However, this clustered organization appears to be cell-type dependent and possibly linked with the specialized functions that each cell type exerts. Indeed, we have recently shown that GPI-AP organization is different in fibroblasts and epithelial cells [5] and, for the latter, it is directly dependent on the establishment of polarity. In fibroblasts, GPI-APs are organized into a mixture of monomers and cholesterol-dependent nanoclusters (∼20–40%) composed of three to four GPI-APs of different species [1,2,4]. In polarized epithelial cells, the apical organization of GPI-APs appears more complex with the coexistence of monomers, homoclusters (∼20–40%) containing 3–5 GPI-APs of same species and larger clusters originating from the coalescence of homoclusters (named heteroclusters because they could contain different GPI-AP species) [5,32,33].

Here, STORM analysis was applied for the first time in polarized epithelial cells to study the spatial distribution of GPI-APs and revealed that at the apical surface of MDCK cells, different GPI-APs (GFP-FR and PLAP) are not randomly distributed, but are confined in areas of ∼60–70 nm radius.

Interestingly, a similar organization has been described for different GPI-APs in fibroblastic Cos-7 cells [15]. Consistently, we found that in CHO cells, another fibroblastic cell type, GPI-APs are organized into clusters of similar radius. This indicates that the dimension of clusters is an intrinsic property of the GPI anchor and is independent of the protein ectodomain or of the cell type. Consistent with this hypothesis, previous data showed that proteins differently anchored to the membrane (either prenylated or transmembrane proteins) reside inside larger clusters (above 60 nm and up to 120–140 nm) compared with GPI-APs [6,15]. Moreover, these findings are in line with a series of single particle tracking studies, showing that the GPI-APs move in transient confinement zones [7,34–37].

In addition, thanks to a theoretical model developed on the basis of our experimental FLIM [5], we could estimate that the size of the heterocluster formed by two homoclusters of equal size is ∼17.4 nm with a distance of ∼1.4 nm between homoclusters. Interestingly, the size estimated by our model is in complete agreement with previous observations based on a combined approach of particle tracking with laser optical trapping, allowing to measure the local viscous drag around proteins. With this approach, Pralle et al. [34] showed that different GPI-APs encounter resistive barriers limiting their diffusion in domains of ∼20–25 nm. As we showed no energy transfer between GPI-AP heteroclusters upon cholesterol depletion [5], these viscous barriers are lost upon same conditions [34], pointing out the critical role of cholesterol in this organization. Strikingly, the fact that the effect of lowering cholesterol in the Pralle experiments is more pronounced for epithelial cells compared with fibroblasts [34] supports further that, in epithelial cells, cholesterol has a major pivotal role at the nanoscale level.

Because it has been demonstrated that homoclusters of GPI-APs are required for their heteroclustered organization [5], STORM data also provide, even indirectly, that different homoclusters are in the same confinement area. In addition, by combining the dSTORM data with the theoretical model, we could resolve that at the apical surface of polarized MDCK cells GPI-APs are constrained into clusters/domains of average 70 nm containing an estimated 4–6 homoclusters. Hence, this clearly indicates a hierarchical organization of GPI-APs at the apical surface of polarized epithelial cells. Similarly, in monocytes and fibroblasts, nanoclusters of GPI-APs have been depicted as enriched in defined regions of the plasma membrane called ‘hotspots’ of both cell types (using homo-FRET and near-field scanning optical microscopy) [3,28].

One interesting open question is why homoclusters do not mix with heteroclusters in epithelial cells. Previous findings showed that the integrated action of both the protein ectodomain and the GPI anchor mediates GPI-AP clustering in epithelial cells [25,29,32]. Although a ‘permissive ectodomain’ prone to oligomerize is required for GPI-AP clustering [29], GPI anchor has an active role. Indeed, it has been demonstrated that GPI-APs (GFP-FR and GFP-PrP) having the same permissive ectodomain, GFP, and different GPI-attachments signals have different ability to cluster [25]. However, it is unknown the precise mechanism by which GPI anchor could act. One possibility is that different GPI-attachment signals lead to different GPI anchors, which have different affinities for lipid domains or modulate the association of the resulting GPI-AP with different lipid domains. The fact that GPI-APs with different GPI-attachment signal/GPI anchor (GFP-FR and PLAP) co-immunoprecipitate only in the presence of the chemical cross-linker BS3 [5], while proteins with the same GPI-attachment signal (possibly same GPI anchor) co-immunoprecipitate also in native conditions (Paladino and Zurzolo, unpublished observations), support the latter hypothesis.

**Figure 5.**
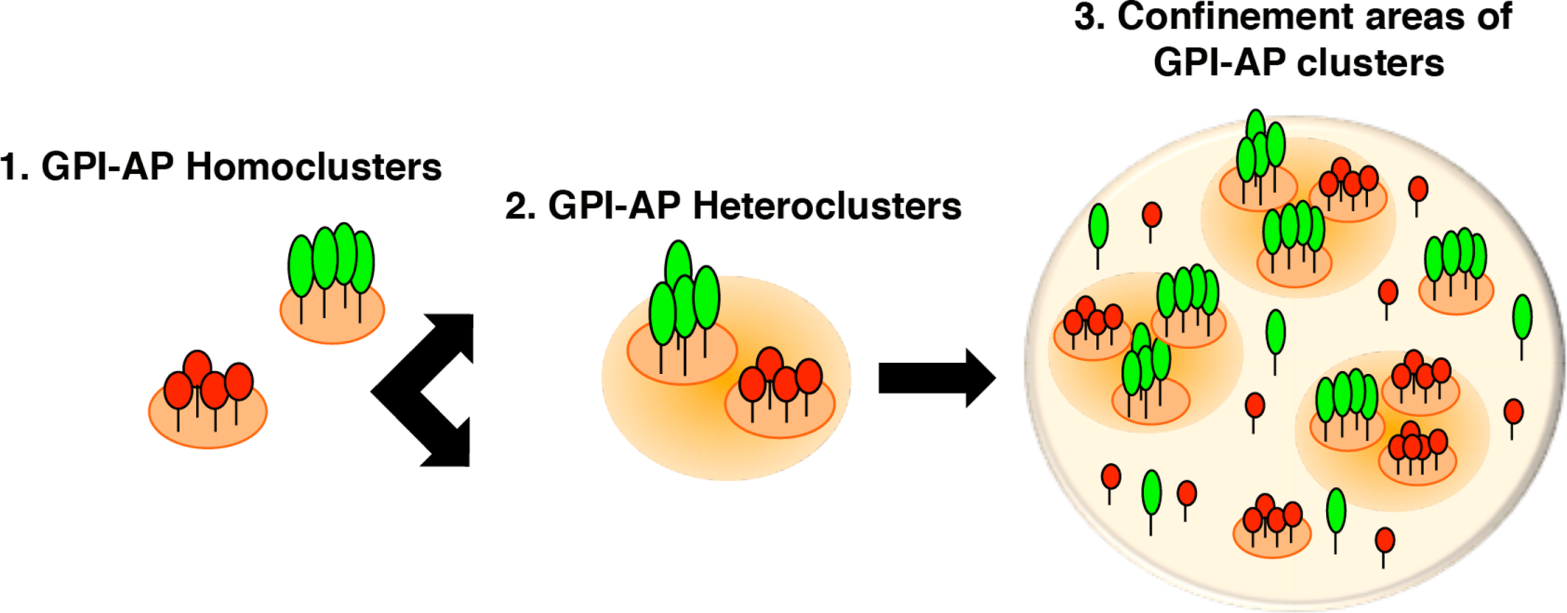
Model of plasma membrane organization of GPI-APs in epithelial cells. At the apical surface of polarized MDCK cells, GPI-APs display three different levels of organization. First, GPI-APs are organized into cholesterol-independent homoclusters of the same GPI-AP species (red and green aggregates) that, in turn, coalesce in cholesterol-dependent heteroclusters. It is conceivable that the affinity of GPI-APs for raft lipids (including sphingolipids and cholesterol) might lead to a local enrichment of cholesterol within confined zones (here depicted as pale orange round domains), which promotes the coalescence of homoclusters excluding monomers. Third, GPI-AP homoclusters are spatially confined.

Our data allow us to resolve three diverse levels of organization at different length scale at the apical surface of polarized epithelial cells (Figure 5): (1) homoclusters of 3–5 GPI-APs (red and green aggregates) held by protein–protein interaction; (2) cholesterol-dependent heteroclusters (size ∼20 nm) derived by coalescence of homoclusters and (3) confinement areas of GPI-AP clusters (size ∼70 nm).

Next question is to decipher what could constrain GPI-APs at the apical surface of epithelial cells: which is (are) the force(s) driving and/or maintaining super-clusters?

Our data exclude a role of cholesterol in the organization of confinement areas of GPI-AP clusters in polarized epithelial cells. Despite similar sizes in epithelial cells and fibroblasts, these areas have different sensitivity to cholesterol. They are cholesterol-dependent in CHO cells, while they are cholesterol-independent at the apical surface of polarized MDCK cells. Although we observed that mevinolin treatment reduces the total cholesterol levels with the same extent in MDCK and CHO cells, we cannot exclude that this treatment could differently affect the plasma membrane cholesterol levels in the two cell types. This could imply that the cholesterol might undergo diverse regulation in the two cell types (e.g. subcellular distribution, enrichment in specific membrane domains) and, in turn, this might affect GPI-AP organization. On the other hand, the different sensitivity to cholesterol could be due to the distinct mechanism of formation of these clusters. While, in CHO cells, monomers of GPI-APs coalesce at the cell surface to organize in clusters, we have previously shown that in polarized epithelial cells, GPI-APs form cluster in the Golgi prior to be delivered to the apical surface [5,11,29]. Because of their formation in the Golgi, apical GPI-APs cluster appears to be independent of the cholesterol modification at the apical surface (Figure 2). Furthermore, epithelial cells exhibit drastic changes in membrane lipid composition during cell polarization as upon epithelial–mesenchymal transition (EMT) induction [38]. In particular, galactosylceramide-sulfate and the Forssman glycolipid, which increased during epithelial polarization and dropdown upon EMT, could contribute to constrain GPI-APs at the apical surface of MDCK cells.

Finally, actin cytoskeleton is an essential player of plasma membrane protein organization. The picket and fence model proposed earlier depicts that transmembrane proteins (associated or not with cholesterol- and sphingolipid-enriched membrane domains) are restrained by their direct interaction with actin cytoskeleton [39–42]. More recently, it was proposed that GPI-AP organization relies on actin cytoskeleton in fibroblasts and that a close relationship between cholesterol and actin exists [3,8]. During polarization, the actin cytoskeleton undergoes a massive rearrangement, compared with non-polarized cells; therefore, it is possible that differences in the cytoskeletal organization might play a role, although our preliminary data (Lebreton, unpublished) seem to confute this hypothesis. Further studies in polarized epithelial cells are required to uncover the molecular components involved in the GPI-AP organization in epithelia. This is of fundamental importance as this appears to be strictly related to the acquisition and the maintenance of the polarized epithelial phenotype that is often challenged in human diseases.

## Abbreviations

dSTORM: direct stochastic optical reconstruction microscopy
EMT: epithelial–mesenchymal transition
FLIM: fluorescence-lifetime imaging microscopy
FRET: fluorescence resonance energy transfer
GPI: glycosylphosphatidylinositol
GPI-APs: GPI-anchored proteins
hA: homocluster labelled by an acceptor
hD: homocluster labelled by a donor
N&B: Number and Brightness
PALM: photo-activated localization microscopy
PC: pair correlation
PFS: Perfect Focus System
PLAP: placental alkaline phosphatase
STORM: stochastic optical reconstruction microscopy
