# Supplementary material for: GPI-anchored proteins are confined in subdiffraction clusters at the apical surface of polarized epithelial cells
Source: Biochem J. 2017 Dec 1;474(24):4075–90. doi: 10.1042/BCJ20170582 (PMC5712066; doi:10.1042/BCJ20170582)
Supplement: Supplementary Figures [file BCJ-474-4075-s1.pdf]

SUPPLEMENTARY INFORMATION

Supplementary Figure legends

Figure S1: Controls for PC-PALM analysis

PC-PALM analyses of STORM experiments performed at the apical surface of fully polarized MDCK cells expressing various proteins: GFP-FR, p75-GFP, monomeric GFP-FR (mGFP-FR) and monomeric GFP-uPAR (mGFP-uPAR).

(A) PC-PALM data and fits. Black dots are pair-correlations computed from the STORM localization data in a single 4x4 μm region. Blue curves are model fits assuming randomly distributed single molecules (equation [1] in Experimental procedures) with a localization precision of σ_s= 22 nm, corresponding to an estimated STORM resolution of ~ 50 nm (see Experimental procedures). Red curves are model fits assuming a clustered organization of molecules.

(B) Histograms show the distribution of mean squared errors between the data and the fitted models for *n* distinct regions of 4x4 μm obtained from *m* independent experiments and an average of *k* cells per experiment. The blue histogram corresponds to the random model, the red histogram corresponds to the clustered model. Overlaps between the two histograms appear in grey. A Kolmogorov-Smirnov test is used to compare the two error distributions and assess if the clustered model (equation [2] in Experimental procedures) provides a significantly better fit to the data than the random model. The resulting p-value is reported in each case and does not reveal significantly different distributions (p>0.1). This indicates that the data are equally well fit by a random model than a more complex clustered model.

(C) Violin plots show the distribution of 2.354*σs obtained by fitting the random model (1) to all 4x4 μm regions in each condition. The median values (red lines) provide an estimate of the STORM image resolution of ~50 nm.

Figure S2: Simulation of random and clustered distribution

Pointillist images (upper panels) and corresponding pc-PALM curves (lower panels) of simulated random (left panels) and clustered (right panels) molecular distribution.

While pointillist images are very similar for random and clustered distribution, pc-PALM analysis shows that the two molecular distributions are different. Indeed, in the case of simulated random distribution (left panel) random model (blue curve) fits better than the clustered model (red curve) the pc-PALM curve (circles), the contrary in the case of simulated clustered distribution (right panel).

Figure S3: Evaluation of protein diffusion in STORM experiments

Boxplots show evaluation of the clusters size in MDCK GFP-FR experiments for the full set of frames (1-30,000) or by selecting either the first 10,000 frames or the last 10,000 frames. The cluster sizes were measured from 42 pc-PALM curves (corresponding to 42 regions of size 4 μm x 4 μm). Note that there is no difference in the cluster size of GFP-FR when we considered either the whole set of images or the first or last 10,000 frames. This indicates that potential diffusion of molecules during acquisition time has a negligible influence on cluster size.

Figure S4: Evaluation of temporal appearance of localizations for random and clustered distributed proteins in STORM experiments

Colour coded localization images reflecting the time frames of detected localizations for p75-NTR and PLAP (in MDCK and CHO cells, respectively). To generate this coloured image, we first merged localizations occurring in consecutive frames within a radius of 100 nm into a single localization, in order to reduce the apparent clustering due to repeated detection of the same molecule. However, our molecules typically blink until the fluorophore bleaches permanently and this can happen multiple times. Therefore, each of these ON times could results as distinct pixel because of localization errors and consequently multiple ON/OFF switching events can lead to

apparent cluster of pixels. To avoid this, we implemented 2D colour coding in which pixels are characterized by both hue (from the average of the frame number of localizations within that pixel) and saturation (from the standard deviation of these frame numbers). Early time frames correspond to blue/violet hue, and later time frames to red hue. Saturated colours (left of the colour bar) indicate that localizations occurring within the pixel are temporally close, whereas unsaturated (whitish) colours (right of colour bar) indicate that localizations occurred at different times during STORM imaging. Because multiple fluorophores within a protein cluster are expected to be stochastically activated at independent time points, they should appear as mixtures of pixels with different colours, or white (unsaturated) pixels, whereas isolated molecules should appear as pixels of homogeneous saturated colour. Moreover, because we observed that the time between the first fluorescent activation of the molecule and its permanent photobleaching is apparently much smaller than the duration of the image sequence acquisition multiple ON/OFF switching events (due to the blinking) will result as pixels of homogeneous saturated colour.

For p75-NTR, the image shows isolated clusters with predominantly homogeneous colours, consistent with pc-PALM analysis, which identified these molecules as having a random distribution (see Figure S1). For PLAP, the image shows predominantly clusters with a mixture of colours and/or less saturated colours, consistent with pc-PALM analysis, which identified this protein as organized in clusters. The pixel size is 36 nm and the scale bar is 2 μm.

Figure S5: Mevinolin treatment reduces the total levels of cholesterol at similar extent in MDCK and CHO cells.

Cholesterol levels of control and mevinolin treated MDCK and CHO cells were measured by colorimetric assay as described in the Experimental procedures. The percentage of cholesterol depletion of treated cells with respect to control cells (set equal to 100) is shown. Results are expressed as mean of three independent experiments ± S.D.

Figure S6: Temporal accumulation analysis of clustering confirms pc-PALM.

Panels show a temporal accumulation analysis on the data shown in Figure 3, as described in Spahn et al. [30, 31]. The normalized localization density in cluster masks, ρ/ρ_0 is plotted as function of the fraction of clustered vs total area, η. Dots show experimental data points, with each color corresponding to a distinct 4 μm x 4 μm region. The red curves show the function expected for a random (non clustered) distribution of molecules. Upon cholesterol depletion (right), the data are close to the red curve, indicating a random distribution of molecules, confirming the pc-PALM analysis of these data. In control conditions (left), the data deviate from the red curve, suggesting a clustering of molecules, also confirming the pc-PALM analysis.

Figure S7: Cholesterol addition does not affect the PLAP organization at the surface of CHO cells

Representative image (4x4 µm area) of STORM localizations of PLAP upon cholesterol addition at the surface of CHO cells is shown. Number of localizations of PLAP is 42236 +/- 28657 (mean +/- standard deviation). Pc-PALM analysis of PLAP is shown as in Figure 1. The pair correlation data are fitted significantly better with a clustered model than a random model ($p<10^{-17}$), revealing a clustered organization of PLAP in these conditions (middle panels). The distribution of calculated PLAP cluster sizes is shown as violin plot, with mean and median as indicated (lower panel).

Figure S8: FLIM analysis

FLIM analysis was performed in MDCK cells after 4 days in culture as previously described in ref. 5. Histograms of GFP-FR lifetime (ns) alone (blue bars) or in combination with mCherry-PLAP (red bars) are shown. Experiments were performed 3 times, n>35 cells. Error bars, ± SD. *, p<0,0001.

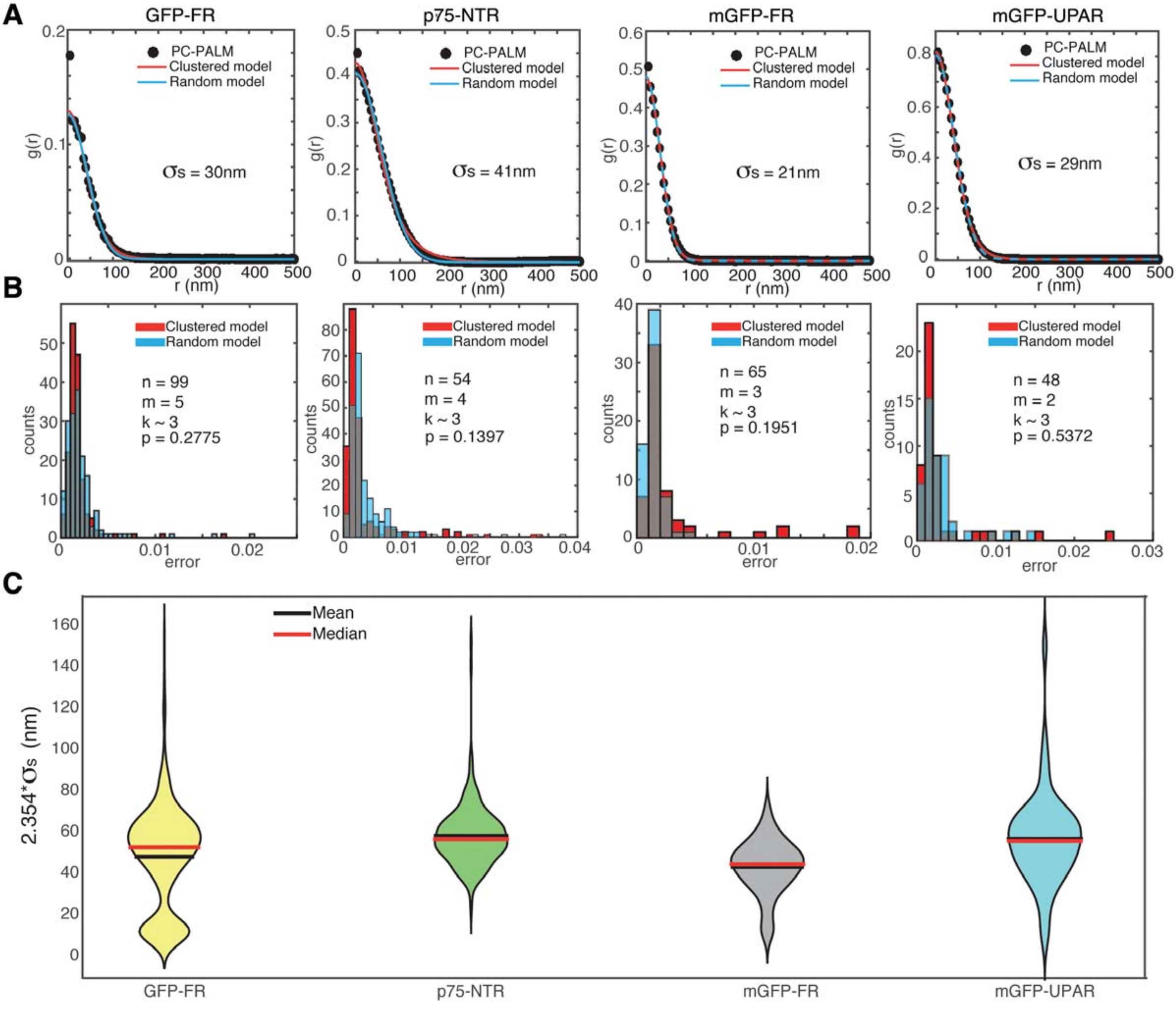
A
GFP-FR
p75-NTR
mGFP-FR
mGFP-UPAR
PC-PALM
Clustered model
Random model
g(r)
r (nm)
σs = 30nm
σs = 41nm
σs = 21nm
σs = 29nm
B
counts
error
n = 99
m = 5
k ~ 3
p = 0.2775
n = 54
m = 4
k ~ 3
p = 0.1397
n = 65
m = 3
k ~ 3
p = 0.1951
n = 48
m = 2
k ~ 3
p = 0.5372
C
Mean
Median
2.354*σs (nm)
GFP-FR
p75-NTR
mGFP-FR
mGFP-UPAR

Figure S1

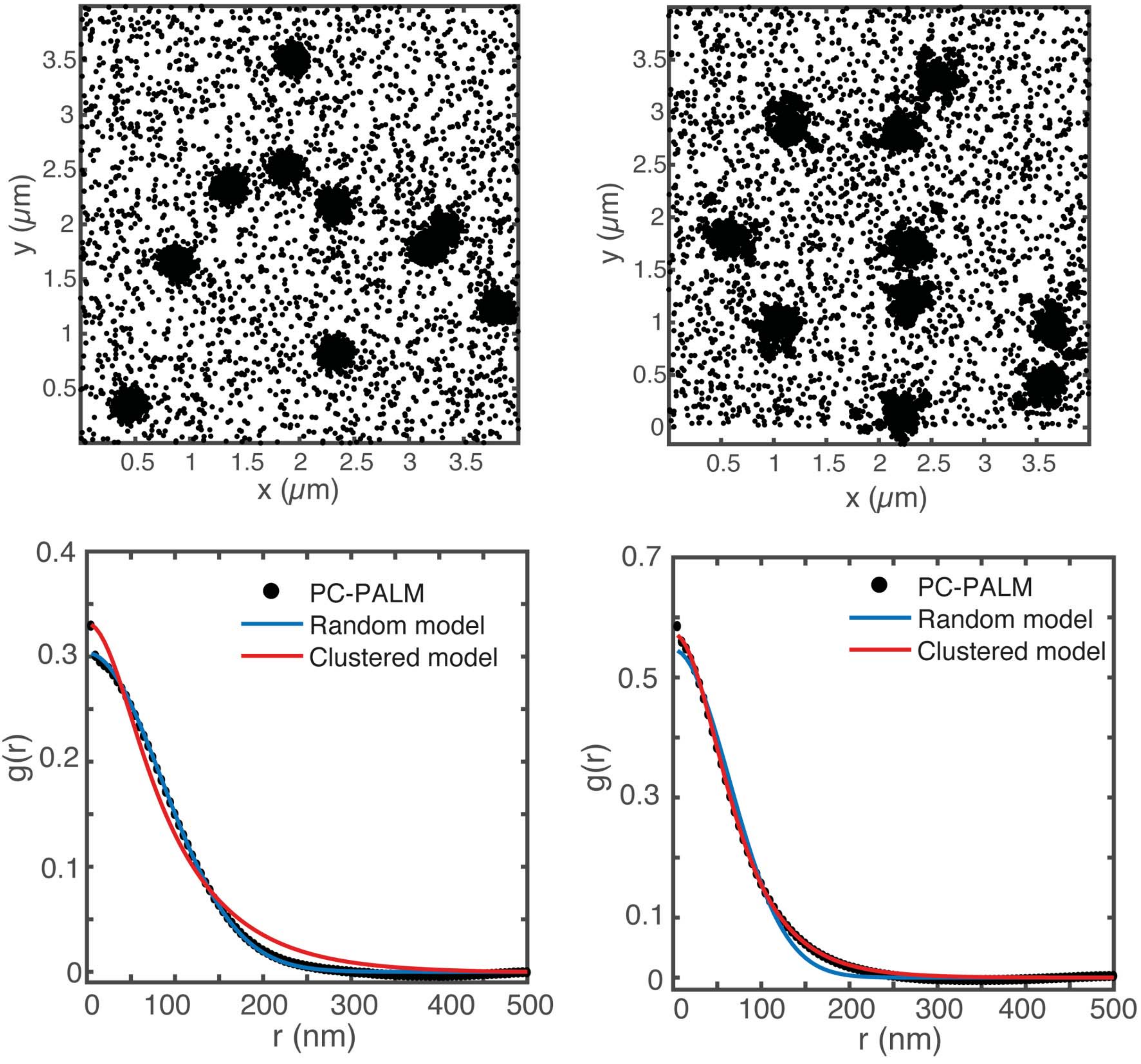

Figure S2

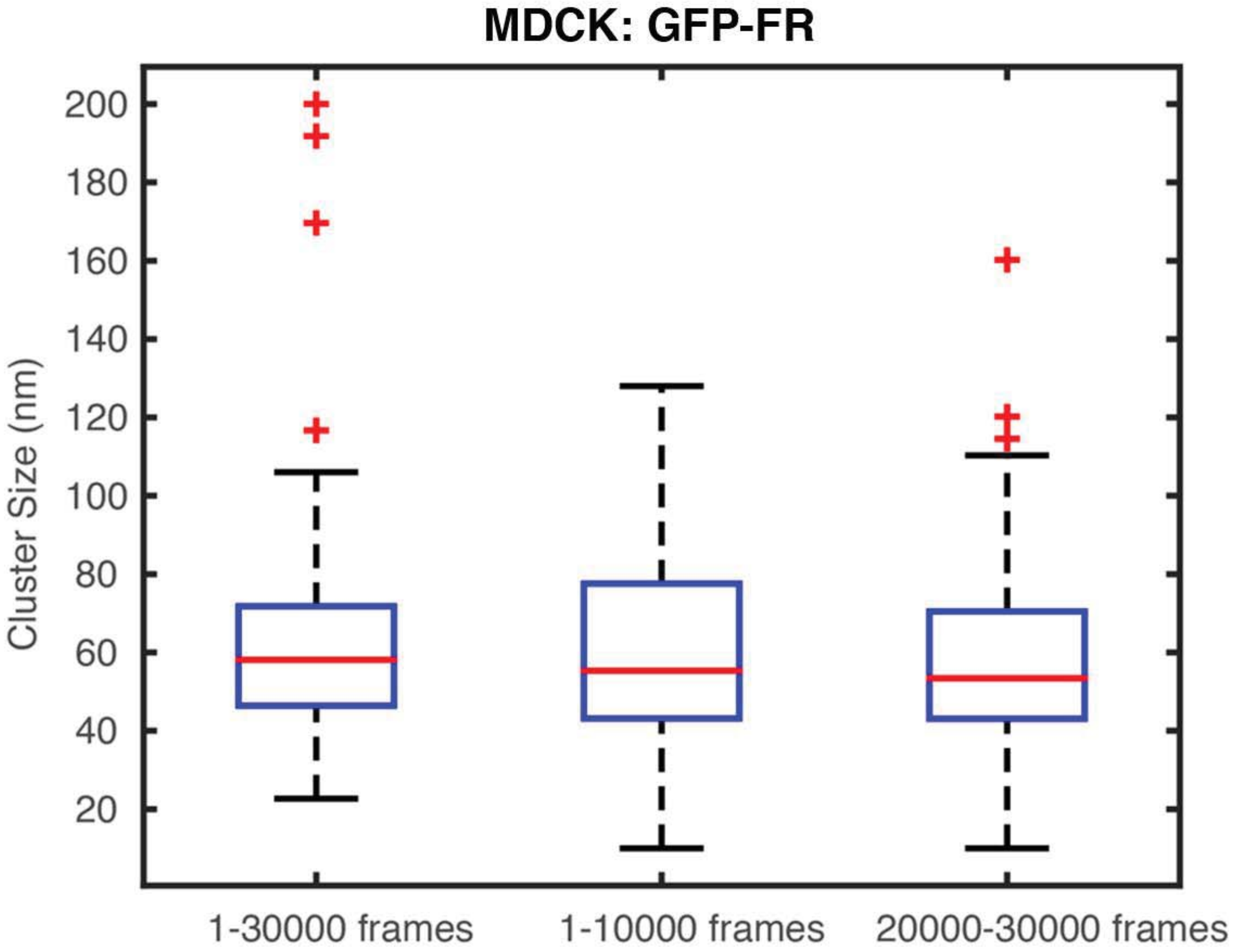
MDCK: GFP-FR
Cluster Size (nm)
200
180
160
140
120
100
80
60
40
20
1-30000 frames
1-10000 frames
20000-30000 frames

Figure S3

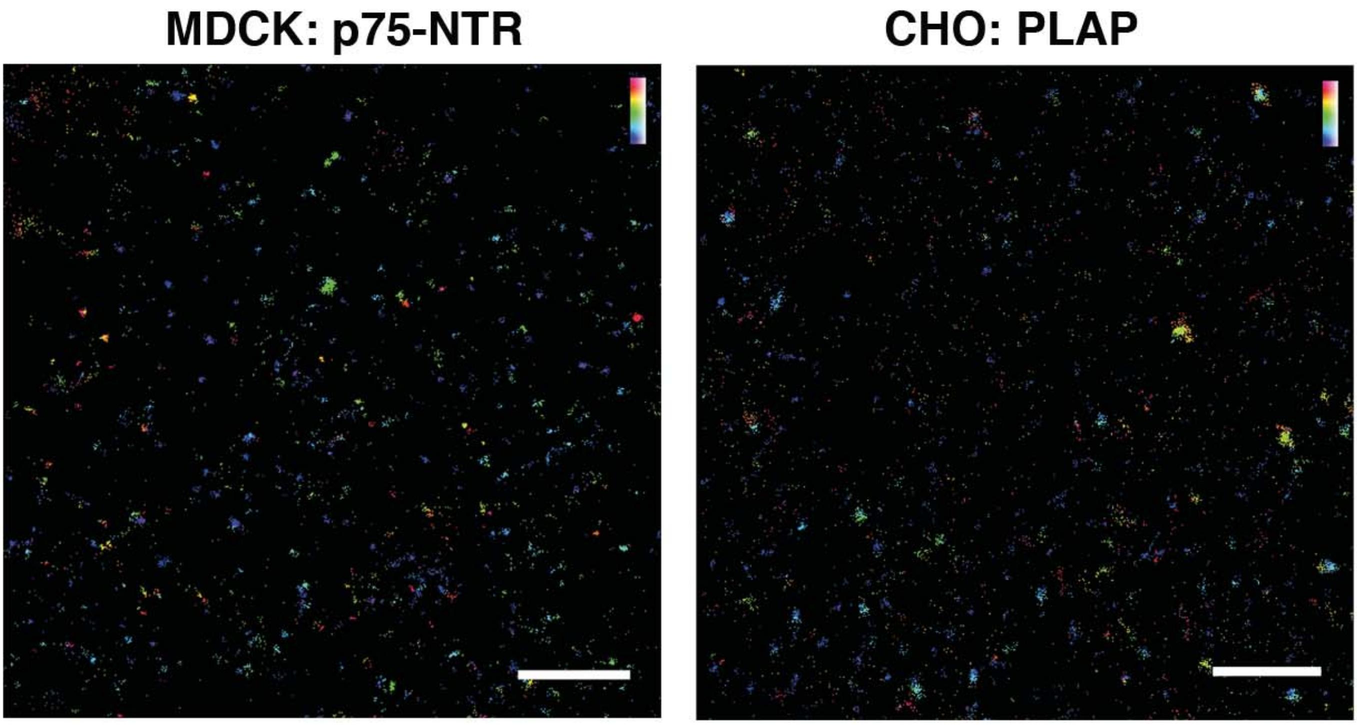

Figure S4

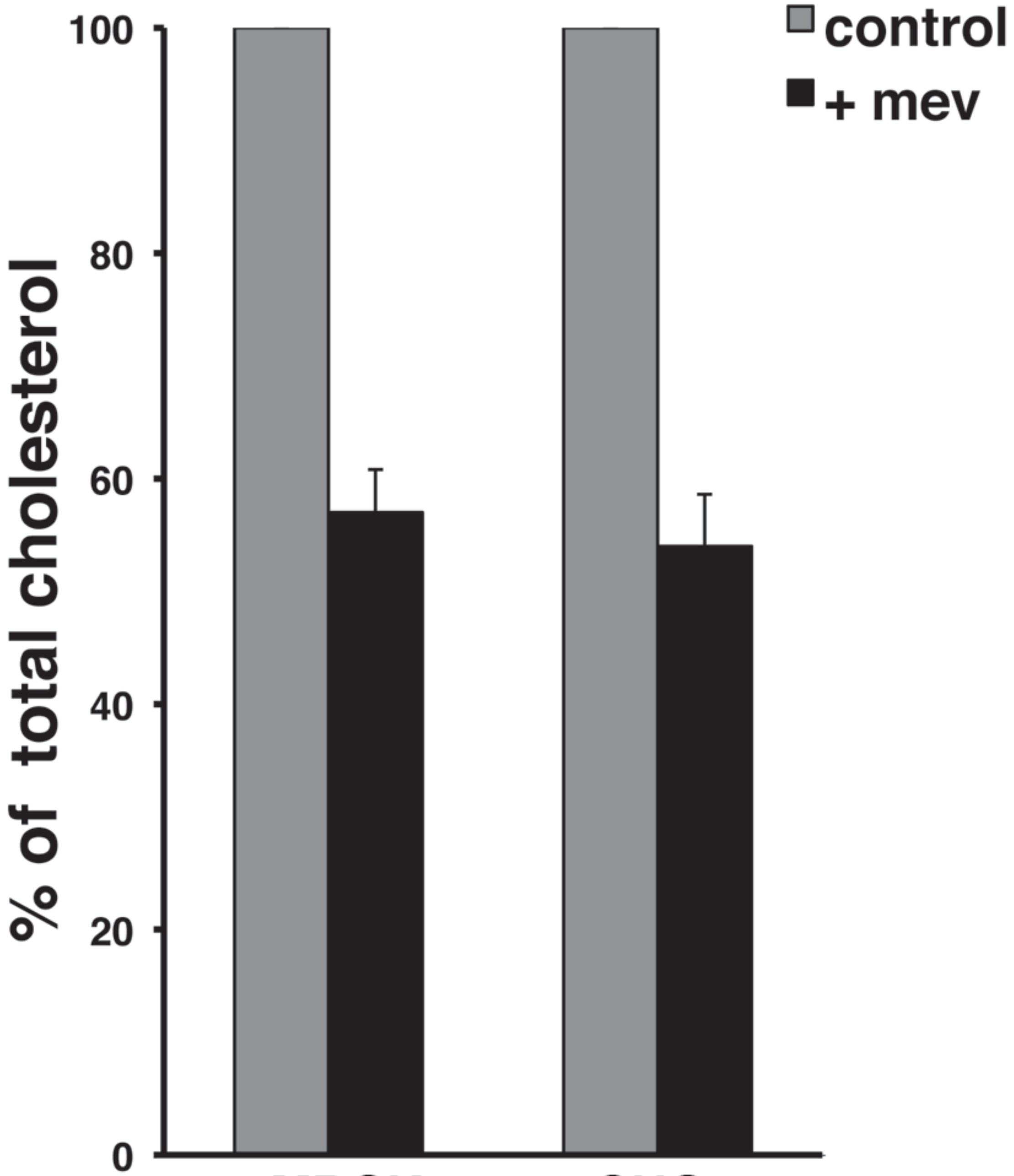

Figure S5

CHO: PLAP

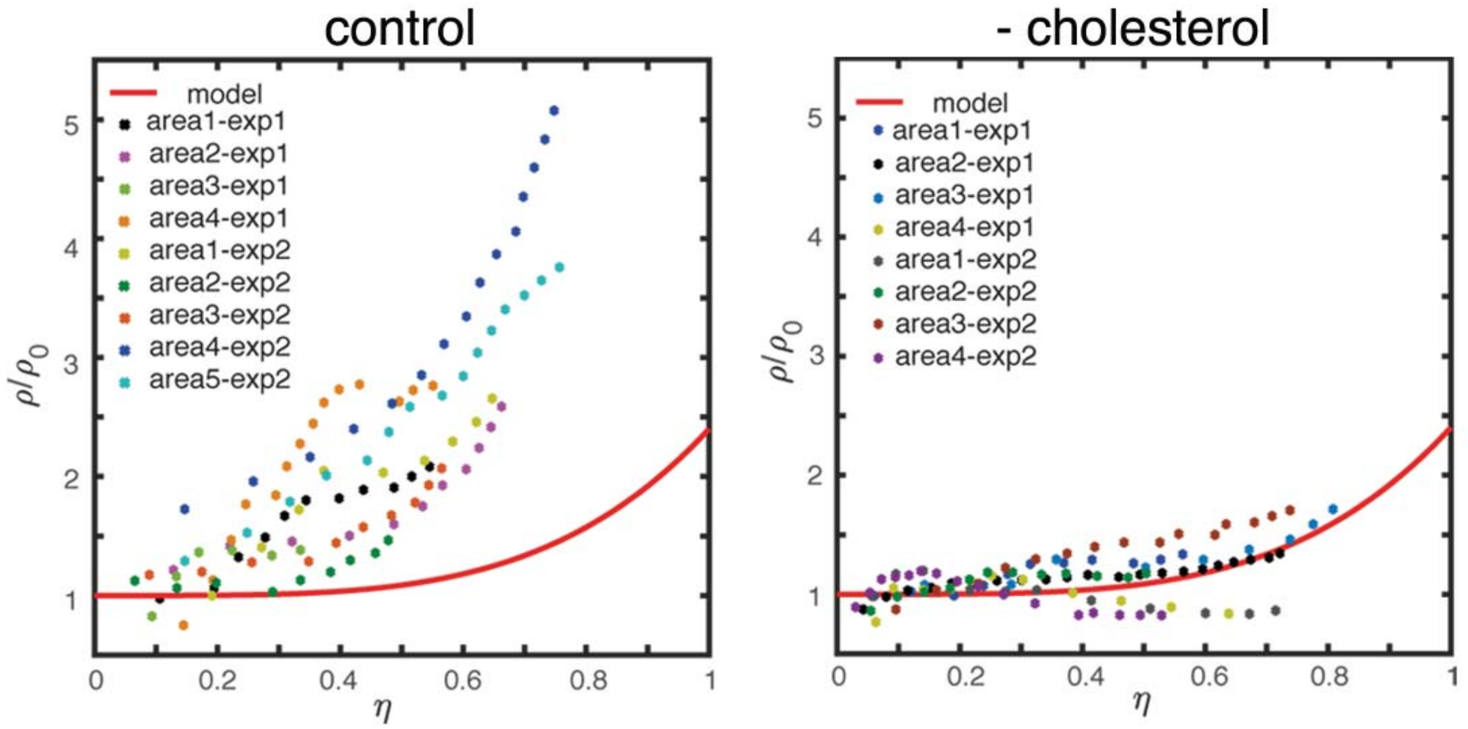

Figure S6

CHO: PLAP + cholesterol

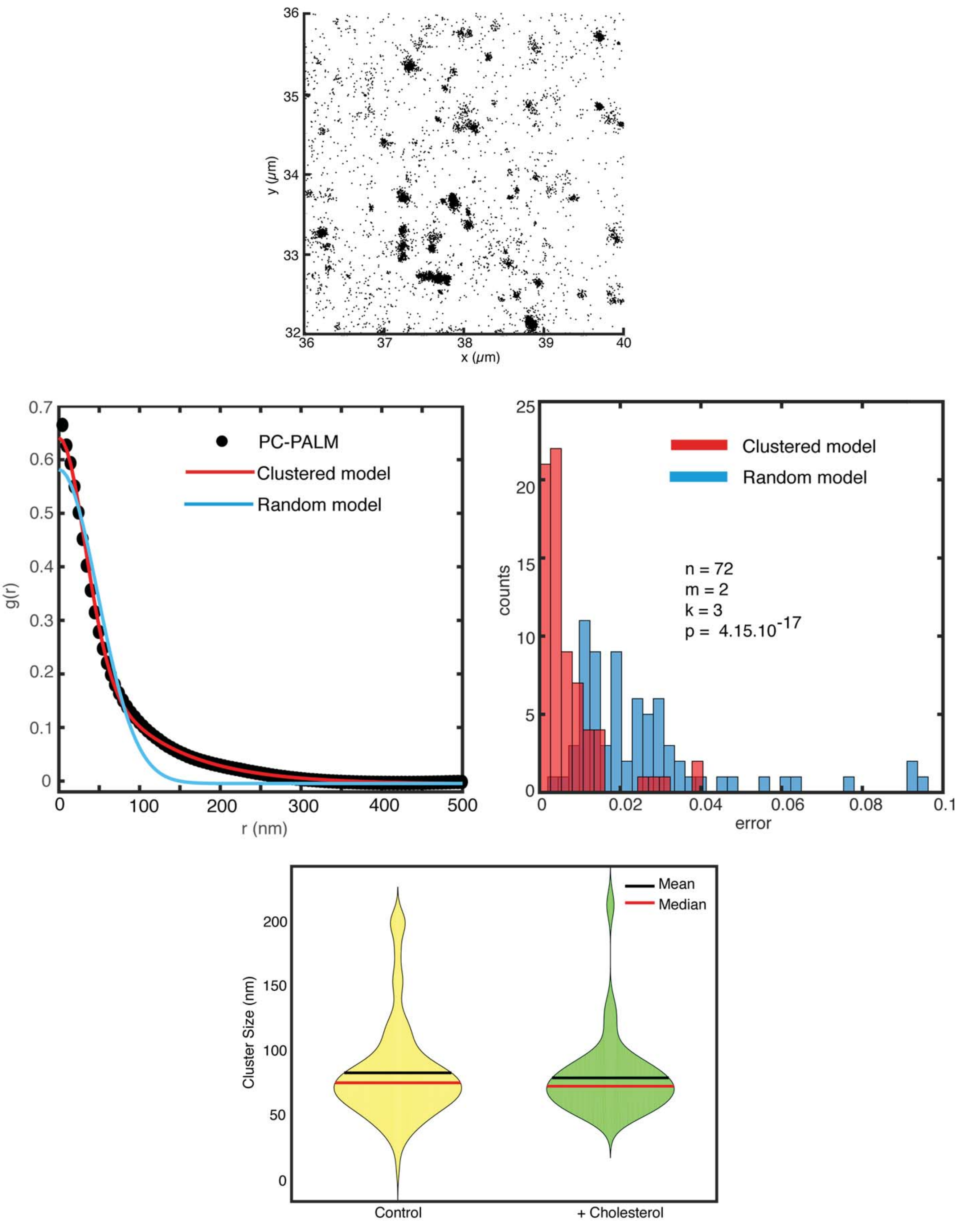

Figure S7

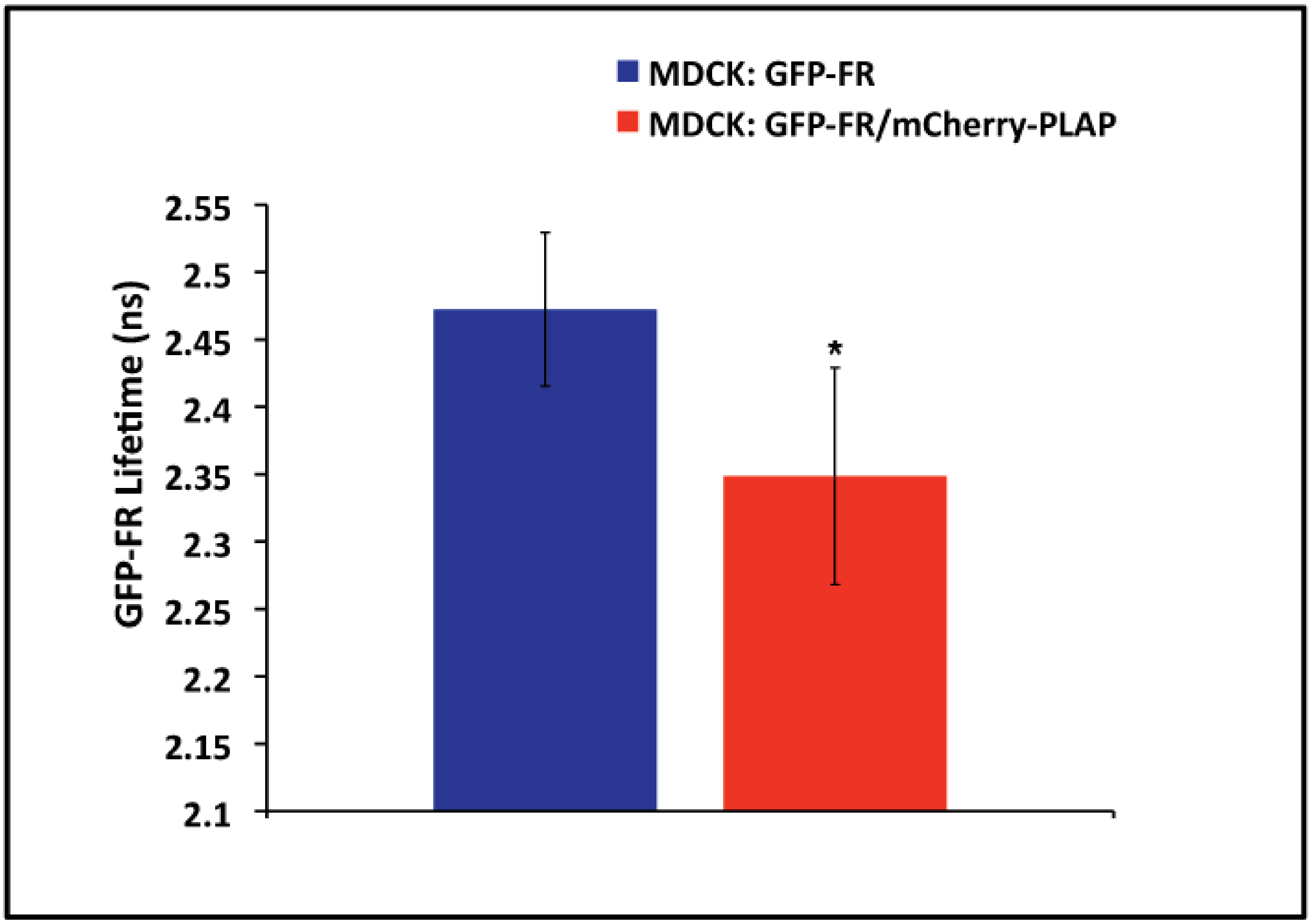
MDCK: GFP-FR
MDCK: GFP-FR/mCherry-PLAP
GFP-FR Lifetime (ns)
2.55
2.5
2.45
2.4
2.35
2.3
2.25
2.2
2.15
2.1
*

Figure S8
